# Evaluation of the self-inflicted violence surveillance system and characterization of reported cases in the Eastern health macro-region of Minas Gerais, 2019-2023

**DOI:** 10.1590/S2237-96222025v34e20240754.en

**Published:** 2025-10-27

**Authors:** Cristian Kelly Costa Santos, Paulo Filipe Silva Siqueira, Waneska Alexandra Alves, Sibele Nascimento Aquino

**Affiliations:** 1 Universidade Federal de Juiz de Fora, Governador Valadares, MG, Brazil

**Keywords:** Health Information Systems, Public Health Surveillance, Suicide, Attempted, Violence, Epidemiology, Descriptive, Sistemas de Información en Salud, Vigilancia en Salud Pública, Intento de Suicidio, Violencia, Epidemiología Descriptiva

## Abstract

**Objectives:**

To analyze the epidemiological characteristics of self-inflicted violence notifications among individuals aged 10 years or older residing in municipalities of the Eastern health macro-region of Minas Gerais from 2019 to 2023, and to evaluate the surveillance system for this condition through the attributes of data quality, representativeness, timeliness, and acceptability.

**Methods:**

This was a cross-sectional study with a descriptive approach, which analyzed the epidemiological profile of self-inflicted violence notifications and the attributes of data quality, representativeness, timeliness, and acceptability, in accordance with the guidelines of the United States Centers for Disease Control and Prevention.

**Results:**

A total of 2,817 cases of self-inflicted violence were reported in the macro-region, 69.7% (1,964) of which occurred among women, with a median age of 25 years. Data quality was classified as good (acceptable duplication rate: 1.9%; moderate consistency: 83.5%; and moderate completeness: 70.9%). Representativeness was considered moderate, based on the completeness of victim profiles (70.1%), time of occurrence (100.0%), and place of occurrence (63.7%). Timeliness was inadequate for 24-hour reporting (52.1%), data entry within seven days (24.8%), and same-day case closure (34.2%). System acceptability was assessed as unacceptable due to low timeliness and unsatisfactory completeness of key variables (68.4%).

**Conclusion:**

Weaknesses were identified in the completion of notification forms and delays in case identification and reporting, rendering the system unacceptable. It is expected that strategies will be strengthened to improve the surveillance of self-inflicted violence, with a focus on prevention efforts.

## Resumen

**Objetivos:**

Analizar las características epidemiológicas de las notificaciones de violencia autoinfligida en personas de 10 años o más, residentes en municipios de la macrorregión sanitaria Este de Minas Gerais en el periodo 2019-2023, y evaluar el sistema de vigilancia de la agresión mediante los atributos de calidad de los datos, representatividad, oportunidad y aceptabilidad.

**Métodos:**

Se trata de un estudio transversal de enfoque descriptivo, que analizó el perfil epidemiológico de las notificaciones de violencia autoinfligida y los atributos de calidad de los datos, representatividad, oportunidad y aceptabilidad, de acuerdo con las directrices de los Centros para el Control y la Prevención de Enfermedades de los Estados Unidos.

**Resultados:**

Se notificaron 2817 casos de violencia autoinfligida en esta macrorregión, de los cuales el 69,7 % (1964) correspondieron a mujeres, con una mediana de edad de 25 años. La calidad de los datos se clasificó como buena (duplicidad aceptable: 1,9 %; consistencia regular: 83,5 %; y completitud regular: 70,9 %). La representatividad fue moderada, teniendo en cuenta la completitud del perfil de las víctimas (70,1 %), el tiempo (100,0 %) y el lugar de la ocurrencia (63,7 %). La oportunidad fue inadecuada para la notificación en 24 horas (52,1 %), la introducción de datos en un plazo de siete días (24,8 %) y el cierre en el mismo día (34,2 %). La aceptabilidad del sistema se evaluó como no aceptable, debido a la baja oportunidad y a la completitud insatisfactoria de las variables esenciales (68,4 %).

**Conclusión:**

Se identificaron deficiencias en la cumplimentación de las notificaciones y falta de oportunidad en la identificación y notificación de los casos, lo que hace que el sistema no sea aceptable. Se espera intensificar las estrategias para mejorar la vigilancia de la violencia autoinfligida, dirigiendo los esfuerzos hacia su prevención.

**Ethical aspects**

This research respected ethical principles, having obtained the following approval data:

Research Ethics Committee: Universidade Federal de Juiz de Fora

Opinion number: 6585259

Approval date: 15/12/2023

Certificate of Submission for Ethical Appraisal: 74689523.3.0000.5147

Informed Consent Form: Dismissed, as this is secondary data, with exemption granted by the ethics committee.

## Introduction 

Self-inflicted violence is manifested daily as minor or severe self-injury or self-mutilation, attempted suicide, and completed suicide (1). It stands out as a significant public health issue due to the magnitude of harm caused to victims, their families, and society as a whole (2). This condition has been subject to mandatory reporting in Brazil since 2011 (3) and immediate notification since 2014 (4). 

An increase in notifications and deaths due to self-inflicted violence has been observed both in Brazil and globally. In the country, notifications, which include suicide attempts, increased by 743.0% from 2011 to 2019 (5). This rise was particularly evident among adolescents in the school setting, with a 2,800.0% increase between 2011 and 2018 (6). From 2014 to 2018, a 19.0% increase in deaths was recorded (7). Suicide mortality also rose among adolescents of both sexes, with an 81.0% increase between 2010 and 2019 (8).

It is crucial to have a robust and resilient surveillance system that enables consistent analysis of morbidity and mortality related to this condition, ensuring rigorous and systematic data collection, analysis, and information sharing to fulfill the system’s intended goals (9). The objectives of the system, namely, timely identification and notification of cases, follow-up of victims through the healthcare network, and analysis of victim profiles and risk factors, must be achieved to support policymakers in developing and improving public policies to address this challenge (10).

Considering the importance of health surveillance systems, periodically evaluating them is essential, as it allows for the monitoring of diseases and conditions relevant to public health, besides enhancing the use of public resources, leading to a more effective and efficient system. It also allows for assessing whether the surveillance of a disease, condition, or health event is meeting the objectives for which it was established. System evaluation is based on attributes that reflect data robustness and reliability, organized into two categories: quantitative (sensitivity, positive predictive value, representativeness, and timeliness) and qualitative (simplicity, flexibility, stability, data quality, and acceptability) (9). 

This study is justified by the relevance of self-inflicted violence in both national and local contexts, with an increase in cases across all age groups and social strata. Thus, understanding how the attributes of the self-inflicted violence surveillance system function in the Eastern macro-region of Minas Gerais is essential to ensure effectiveness and accuracy in prevention and intervention efforts. 

This study aimed to analyze the epidemiological characteristics of self-inflicted violence notifications among individuals aged 10 years or older residing in municipalities in the Eastern health macro-region of Minas Gerais from 2019 to 2023, and to evaluate the surveillance system for this condition using the attributes of data quality, representativeness, timeliness, and acceptability.

## Methods 

### Study design

This was a cross-sectional study with a descriptive approach that analyzed the epidemiological profile of self-inflicted violence notifications and the attributes of data quality, representativeness, timeliness, and acceptability, in accordance with guidelines from the United States Centers for Disease Control and Prevention (CDC) (9). Notifications of residents in the Eastern macro-region of Minas Gerais, recorded in the Notifiable Health Conditions Information System (Sinan) between 2019 and 2023, were analyzed. It is important to note that the study period was defined based on data analysis before, during, and after the COVID-19 pandemic.

### Setting 

Minas Gerais is a federal state located in southeastern Brazil. It has 16 health macro-regions. It is the state with the most significant number of municipalities and the second most populous in the country (11). The Eastern health macro-region of Minas Gerais includes 51 municipalities, grouped into four micro-regions whose hub municipalities are Governador Valadares, Mantena, Resplendor, and Peçanha/São João Evangelista/Santa Maria do Suaçuí (12). In 2022, the region’s population was 643,031. Excluding Governador Valadares, the average population per municipality is 7,717, indicating that most are small-sized municipalities. Governador Valadares is the seat of the macro-region, hosts the most complex health services, and has a population of 257,172 (11).

### Data sources

Secondary nominal data from Sinan were used, referring to individuals aged 10 years or older. These were provided by the Governador Valadares Regional Health Superintendency in March 2024, which ensured access to real-time, updated data. This regional unit is part of the Minas Gerais State Health Department and is responsible for the health system’s organizational structure within the macro-region. 

Sinan operates in a decentralized manner at the municipal level. All health services were reporting units, and their notifications were entered into the national surveillance database for this condition. Only records notified as suspected or confirmed self-inflicted injury (suicide attempt or self-mutilation) were included, in accordance with current guidelines from the Ministry of Health (1). The study population was defined considering the difficulty of determining the intentionality of self-injury in children under 10 years of age (13). 

### Study variables

In the descriptive phase, the following sociodemographic variables were analyzed: sex (male, female); age group in years (10–19, 20–34, 35–49, 50–64, 65–79, ≥80), ace/skin color (White, Black/Brown, Asian, Indigenous, unknown/blank), education in years of schooling (<4; 4–7; 8–10; ≥11, unknown/blank), marital status (single, married/common-law marriage, widowed, separated/divorced, not applicable, unknown/blank); sexual orientation (heterosexual, homosexual, bisexual, not applicable, unknown/blank); gender identity (transvestite, transgender woman, transgender man, not applicable, unknown/blank); and presence of any kind of disability/disorder (yes, no, unknown/blank). 

For the analysis of the occurrence, the following variables were assessed: place of occurrence (home, public place, other, unknown/blank), history of prior episodes (yes, no, unknown/blank), means of aggression (hanging, poisoning/intoxication, sharp/piercing object, physical force/beating, blunt object, hot substance or object, threat, firearm, other) and referral to healthcare services (yes, no, unknown/blank). The variables in this study were defined based on their epidemiological relevance, as recommended by the Ministry of Health (14).

For the evaluative approach, the following attributes were analyzed (Table 1).

**Table 1:** Parameters used to evaluate the attributes of data quality, representativeness, timeliness, and acceptability

Attribute	Items for evaluation	Classification	Calculation formula	Overall classification
**Data quality**	Duplicates	Acceptable (A) <5.0%, Not acceptable (NA) ≥5.0%	Percent average=n duplicates*100/n notifications	Excellent (A duplicates, E completeness, E consistency); Good (NA duplicates, E/B/Re completeness, E/Re consistency; A/NA duplicates, B/Re/R completeness, E/Re/B consistency; A/NA duplicates, E/B/Re/R/MR completeness, Re/R inconsistency); Poor (NA duplicates, R/MR completeness, B inconsistency)
	Completeness	Excellent (E)≥95.0%; Good (B) 90.0–94.9%; Regular (Re) 70.0–89.9%; Poor (R) 50.0–69.9%; Very poor (MR)<50.0%	Percent average=sum of completeness percentages of variables/n variables	
	Consistency	Excellent (E)≥90.0%; Regular (Re)≥70.0 e <90.0%; Low (R)<70.0%	Percent average=sum of consistency percentages of variables/n variables	
Representativeness	Person (victim profile)	Representative (R) ≥70.0%; Poorly representative (PR) <70.0%	Sum of completeness percentages of variables/n variables	Representative (R=person, time, and place); **Moderate representativeness** (two R results+one PR); **Poor representativeness** (PR=person, time, and place, or one R and two PR)
	Time (date of occurrence)		Percentage of completeness of the occurrence date variable	
	Place (place of occurrence)		Percentage of completeness of the place of occurrence variable	
Timelines	Notification within 24 hours of the date of occurrence.	Timely (T) ≥90.0% Untimely (U): <90.0%	N notifications within 24h*100/n notifications	Timely (notification within 24h, data entry within seven days, and case closure on the same day=T); Timely (two T and one U); Untimely (notification within 24h, data entry within seven days, and case closure on the same day=U or one T and two U)
	Data entry within 7 days		N data entry within seven days*100/n notifications	
	Case closure on notification day		N notifications closed on the same day*100/n notifications	
Acceptability	Timely notification	Acceptable (A): ≥70.0% Not acceptable (NA): <70.0%	N notifications within 24h*100/n notifications	Acceptable (timely notification and completeness percentage meet the acceptability standard=A); Acceptable (one A and one NA=A); Not acceptable (both timely notification and completeness=NA)
	Completeness of eight variables		Percent average=sum of completeness percentages of variables/n variables

- Data quality referred to the presence of valid, complete, and accurate information, considering three dimensions: completeness, consistency, and absence of duplicate entries (9).
- Completeness referred to the proportion of required and essential fields in the notification form filled out with valid information, i.e., without blank or unknown responses. Sociodemographic and occurrence variables, including occupation and pregnancy status, were evaluated. The parameters used to assess completeness ranged from “excellent” to “very poor” (15).
- Consistency referred to the logical conformity between fields or data sets. Parameters ranged from “excellent” to “low” consistency (16). The following combinations of variables were evaluated: male sex and pregnancy field marked as “not applicable”; date of birth compatible with the victim’s age; self-inflicted injury and means of aggression other than threat; sex of perpetrator different from sex of victim; degree of kinship between perpetrator and victim “the person themselves”; self-inflicted injury and type of violence with option marked as “other” with the additional information of self-inflicted violence, or any other term or expression indicating this type of violence; date of notification equal to or later than the occurrence
- Duplication was defined as the same case being registered more than once in the surveillance system—duplicate analysis involved identifying and removing repeated records. Individuals with the same full name, date of birth, sex, age, mother’s name, date and place of occurrence, and observation field were considered the same person (1). The criterion for acceptable duplication was the one defined by Abath et al. (17).
- Representativeness referred to the extent to which the collected data could describe the condition in terms of person, time, and place (9). To evaluate this attribute, variables related to victim profile (sex, age, education, race/skin color, marital status, gender identity, and sexual orientation), year, and place of occurrence were considered.
- Timeliness referred to the time interval in which information about the event became available to those who needed it in order to initiate appropriate control actions (9). Notifications of self-inflicted violence to municipal epidemiological surveillance were expected within 24 hours of the occurrence, and Sinan data entry was to occur within seven days. Cases were considered closed when the notification form was completed (1,4,7).
- Acceptability was defined as the degree to which professionals and institutions participated in the surveillance system and were willing to collaborate and use the system to implement immediate actions to mitigate the risk of recurrence (9,18). Timely notification (within 24 hours) and the completion of relevant information for case management were evaluated, including: race/skin color, education, disability/disorder, sexual orientation, gender identity, history of prior episodes, place of occurrence, means of aggression, and referral to healthcare services. The system was considered acceptable when the average of timeliness and completeness indicators was equal to or greater than 70.0% (19).

### Bias 

To minimize potential selection and information biases, only records in Sinan that were classified as suicide attempts or intentional self-harm were included, based on standardized Ministry of Health criteria. Records with incomplete or inconclusive information regarding the intentionality of the event were excluded to ensure the quality and reliability of the data analyzed. To ensure population representativeness and minimize geographic bias, only notifications from municipalities in the Eastern health macro-region of Minas Gerais were included. 

### Statistical methods

Exploratory descriptive analyses were performed using absolute (n) and relative (%) frequencies, along with inferential analyses using the Mann-Whitney U test for age and Pearson’s chi-square test to identify associations between categorical variables of interest within the study population. Analyses were performed using Microsoft Excel and Jamovi software (v.2.4.8), with a significance level of 5.0% (p-value<0.05). After analysis, the data were anonymized and made available in the following repository: https://doi.org/10.48331/scielodata.OGYNWG (20).

## Results 

From 2019 to 2023, a total of 2,873 cases of self-inflicted violence were reported among individuals aged 10 years or older residing in municipalities of the Eastern macro-region of Minas Gerais. After excluding 56 duplicate records (1.9%), 2,817 reports were analyzed (Supplementary Table 1). The time series revealed a decline in notifications during the pandemic in 2020 (n=381) and 2021 (n=393), representing reductions of 40.5% and 38.6%, respectively, compared to 2019 (n=640).

The median age of victims was 25, ranging from 10 to 91 years, with a predominance of young adults aged 10–34 years (69.9%; n=1,968; p-valor<0.001). Most victims were female (69.7%; n=1,964; p-valor<0.001) and self-identified as Black or Brown (74.9%; n=2,111; p-valor=0.071). Regarding the occurrence, differences were found in the place of the incident, with predominance in the variable home (59.7%; n=1,681; p-value<0.001), and in the means of aggression — hanging among men (13.8%; n=118; p-value<0.001) and poisoning among women (62.5%; n=1,228; p-value<0.001) (Table 2).

**Table 2:** Characteristics of self-inflicted violence cases reported in the Notifiable Health Conditions Information System (Sinan), by sex. Eastern health macro-region, Minas Gerais, 2019–2023

Variables	Total n (%)	Male n (%)	Female n (%)	p-value
	2,817 (100.0)	853 ( 30.3)	1,964 ( 69.7)	
Sociodemographic variables				
**Age group** (years)				<0.001
10-19	879 (31.2)	207 (24.3)	672 (34.2)	
20-34	1,089 (38.7)	362 (42.4)	727 (37.3)	
35-49	594 ( 21.1)	168 (19.7)	426 (21.7)	
50-64	194 ( 6.9)	78 ( 9.1)	116 (5.9)	
65-79	52 (1.8)	30 (3.5)	22 (1.1)	
≥80	9 ( 0.3)	8 (0.09)	1 ( 0.1)	
**Race/skin color**				0.071
White	511 (18.1)	130 (15.2)	381 (19.4)	
Black/Brown	2.111 (74.9)	656 (76.9)	1.455 (74.1)	
Asian	37 (1.3)	12 ( 1.4)	25 ( 1.3)	
Indigenous	8 ( 0.8)	2 ( 0.2)	6 ( 0.3)	
Unknown/Blank	150 (5.3)	54 (6.2)	97 ( 4.9)	
**Education level** (years)				<0.001
<4	80 (2.8)	33 (3.9)	47 (2.4)	
4-7	271 (9.6)	75 (8.8)	196 ( 10.0)	
8-10	406 (14.4)	110 (12.9)	296 ( 15.1)	
≥11	372 (13.2)	86 ( 10.6)	286 (14.6)	
Unknown/Blank	1,688 (59.9)	549 (64.4)	1.139 (58.0)	
**Marital status**				0.559
Single	1,152 (40.9)	359 (42.1)	793 ( 40.4)	
Married/Common-law marriage	504 (17.9)	137 (16.1)	367 (18.7)	
Widowed	20 ( 0.7)	4 ( 0.5)	16 ( 0.8)	
Divorced	87 (30.1)	26 ( 3.0)	61 (3.1)	
Not applicable	31 ( 1.1)	10 ( 1.2)	21 ( 1.1)	
Unknown/Blank	1,023 (36.3)	317 (37.1)	706 (35.9)	
**Sexual orientation**				0.167
Heterosexual	1,196 (42.5)	338 (39.6)	858 (43.7)	
Homosexual	50 (1.8)	20 ( 2.3)	30 (1.5)	
Bisexual	19 ( 0.7)	5 ( 0.6)	14 ( 0.7)	
Not applicable	99 ( 3.5)	28 (3.3)	71 (3.6)	
Unknown/Blank	1,453 (51.6)	462 (54.2)	991 ( 50.5)	
**Gender identity**				0.002
Transvestite	6 ( 0.2)	6 ( 0.7)	0 ( 0.0)	
Transgender woman	14 ( 0.5)	4 ( 0.5)	10 ( 0.5)	
Transgender man	0 ( 0.0)	0 ( 0.0)	0 ( 0.0)	
Not applicable	1,208 (42.9)	351 (41.1)	877 ( 43.6)	
Unknown/Blank	1,589 (46.4)	492 (57.7)	1,097 (55.9)	
**Disability or disorder**				0.156
Yes	880 ( 31.2)	268 (31.4)	612 (31.2)	
No	942 (33.4)	262 (30.7)	680 (34.6)	
Unknown/Blank	995 ( 35.3)	323 (37.9)	672 (34.2)	
Occurrence variables				
**Place of occurrence**				<0.001
Home	1,681 (59.7)	447 (52.4)	1.234 (62.8)	
Public spaces	120 (4.3)	50 ( 5.9)	70 (3.6)	
Other	136 (4.8)	67 ( 7.9)	69 ( 3.5)	
Unknown/Blank	880 (31.2)	289 (33.9)	591 ( 30.1)	
**History of prior episodes**				<0.001
Yes	945 (33.5)	220 (25.8)	725 (36.9)	
No	756 (26.8)	266 (31.2)	490 ( 24.9)	
Unknown/Blank	1,116 ( 39.6)	367 (43.0)	749 (38.1)	
**Means of aggression**				
Hanging	194 (6.9)	118 ( 13.8)	76 (3.9)	<0.001
Poisoning/intoxication	1,655 (58.8)	427 (50.1)	1,228 (62.5)	<0.001
Sharp/piercing object	516 (18.3)	140 (16.4)	376 (19.1)	0.188
Physical force/beating	205 (7.3)	73 (8.6)	132 (6.7)	0.181
Blunt object	67 ( 2.4)	20 ( 2.3)	47 ( 2.4)	0.91 8
Hot substance or object	35 (1.2)	9 ( 1.1)	26 (1.3)	0.724
Threat	59 ( 2.1)	17 ( 2.0)	42 (2.1)	0.862
Firearm	18 ( 0.6)	9 (1.1)	9 ( 0.5)	0.144
Other	262 (9.3)	81 (9.5)	181 (9.2)	0.572
**Referral** (**healthcare services**)				0.793
Yes	2,556 (90.7)	770 (90.3)	1,786 (90.9)	
No	233 (8.3)	75 (8.8)	158 (8.0)	
Unknown/Blank	28 ( 1.0)	8 ( 0.9)	(1.0)	

^a^Mann-Whitney U test; ^b^Pearson chi-square test.

Data quality was assessed considering completeness, duplication, and consistency. Completeness was rated as excellent or good for the variables of sex, age, referral to healthcare services, means of aggression, and race/skin color (Table 3). With regard to duplication, 2019 had the lowest percentage of duplicate records (0.9%; n=6), while 2022 had the highest (3.6%; n=23) (Supplementary Table 1). 

**Table 3:** Completeness of fields on the self-harm notification form. Eastern health macro-region, Minas Gerais, 2019–2023

Variables	2019	2020	2021	2022	2023	Total (n=2,817) %	Classification
	(n=640) %	(n=381) %	(n=393) %	(n=613) %	(n=790) %		
Mandatory							
Sex	100.0	100.0	100.0	100.0	100.0	100.0	Excellent
Age	100.0	100.0	100.0	100.0	100.0	100.0	Excellent
Referral to healthcare services	99.7	99.7	100.0	98.5	98.0	99.0	Excellent
Pregnant person	62.3	84.3	77.9	86.0	87.8	79.8	Regular
Place of occurrence	58.8	63.3	58.3	71.1	82.9	68.8	Poor
Sexual orientation	41.1	48.0	38.2	50.1	58.4	48.4	Very poor
Gender identity	32.5	43.8	33.4	51.9	51.4	43.6	Very poor
Essential							
Means of aggression	90.3	97.4	97.3	95.0	96.5	95.6	Excellent
Race/skin color	93.4	97.6	88.5	94.3	97.6	94.7	Good
Marital status	64.0	59.8	52.7	61.3	72.5	63.7	Poor
Disability/disorder	43.8	50.9	54.7	64.8	93.2	64.8	Poor
History of prior episodes	55.5	56.7	52.7	58.4	71.5	60.4	Poor
Education level	35.3	34.4	28.0	41.6	51.6	40.1	Very poor
Occupation	31.6	27.3	21.6	36.3	43.9	34.1	Very poor

Consistency assessed the coherence among seven related variables, with five classified as excellent (male sex and pregnancy field marked as “not applicable”; notification date equal to or later than the occurrence; date of birth consistent with the victim’s age; self-inflicted injury marked as “yes” and degree of kinship between perpetrator and victim marked as “the person themselves”; and a valid, non-threat-related means of aggression) (Table 4). In the overall analysis, the average percentage and classification over the five years assessed were: duplication 1.9% (acceptable); consistency 83.5% (fair); and completeness 70.9% (fair). Data quality was classified as good.

**Table 4:** Consistency between related variables in the self-harm notification form. Eastern health macro-region, Minas Gerais, 2019–2023

Variables	2019	2020	2021	2022	2023	Total (n=2,817) %	Classification
	(n=640) %	(n=381) %	(n=393) %	(n=613) %	(n=790) %		
Male sex and pregnancy field marked as “not applicable”	100.0	100.0	100.0	100.0	100.0	100.0	Excellent
Notification date equal to or later than the occurrence	100.0	100.0	100.0	100.0	100.0	100.0	Excellent
Date of birth consistent with the victim’s age	96.9	96.3	95.4	97.6	97.6	97.0	Excellent
Degree of kinship between perpetrator and victim marked as “the person themselves”	92.2	87.9	90.4	87.4	92.4	90.5	Excellent
Valid, non-threat-related means of aggression	88.0	95.0	97.2	92.7	94.8	93.1	Excellent
Sex of perpetrator different from sex of victim	90.3	86.3	84.7	85.5	90.0	87.9	Regular
Type of violence with additional information on self-inflicted violence	6.7	9.7	6.6	11.3	35.2	16.1	Low

The data evaluation indicated that the system is representative for person-based variables, with an average completeness of 70.1% for variables related to the victim profile, and 100.0% for time (date of occurrence). Regarding place, the system was less representative, with a completeness rate of 63.7%. Thus, system representativeness was considered moderate.

It is worth noting that, in terms of location, considering the municipality of occurrence, the distribution across the macro-region by municipality was 98.0% (n=50), as only one municipality reported no self-inflicted violence during the period. There was an increase in the distribution of occurrences by municipality, with 84.3% (n=43) of municipalities reporting cases in 2019, rising to 94.1% (n=48) in 2023. 

System timeliness was rated as inadequate for case notification within 24 hours, data entry within seven days, and closure on the same day as the notification (Table 5). The median time to case notification was one day (range: 0–4,778 days); for data entry into Sinan, 16 days (range: 0–4,781 days); and for case closure, three days (range: 0–735 days). 

**Table 5:** Assessment of the timeliness of self-inflicted violence cases reported in the Notifiable Health Conditions Information System (Sinan). Eastern health macro-region, Minas Gerais, 2019–2023

Variables	2019	2020	2021	2022	2023	Total	
	(n=640) %	(n=381) %	(n=393) %	(n=613) %	(n=790) %	(n=2,817) %	p-value
Cases reported within 24 hours after occurrence	35.0	50.9	45.8	59.1	64.4	52.1	<0.001
Cases entered into SINAN within seven days after occurrence	16.3	25.5	14.2	32.8	30.6	24.8	<0.001
Cases closed on the same day as notification	28.0	37.0	34.9	32.0	39.6	34.2	<0.001

^a^Pearson’s chi-square test.

The system was also classified as unacceptable regarding the timely identification and reporting of cases, since only 52.1% (n=1,469) were notified within 24 hours. The average completeness percentage for variables considered essential for the care and management of victims was 68.4%. These variables included: race/skin color, level of education, presence of disorder/disability, sexual orientation, gender identity, history of prior episodes, place of occurrence, means of aggression, and referral to healthcare services. 

## Discussion 

The results revealed some strengths and weaknesses of the macro-regional self-inflicted violence surveillance system. It showed good data quality and moderate representativeness; however, it was untimely for all evaluated time-related variables and deemed unacceptable by health professionals, as timely case identification and notification—one of the essential premises of any surveillance system—was not achieved in most cases.

It is worth noting that working with secondary data may present certain limitations, such as underreporting, missing information relevant to the study, and improper completion of notification forms. Other limitations included the evaluation of only four of the nine attributes recommended by the CDC (9) and the COVID-19 pandemic, which led to a significant reduction in the number of notifications during that period. Despite some limitations, the results presented in this paper were robust in offering an overall picture of self-inflicted violence surveillance in the macro-region, with year-by-year data analysis allowing for assessment before, during, and after the COVID-19 pandemic. 

The novel coronavirus pandemic significantly impacted notifications, making underreporting during the pandemic years evident. This period was marked by strict restrictions and the imposition of social isolation measures to control the disease. Health services became overwhelmed and had to prioritize care for patients with more severe conditions, which may have hindered access to some outpatient and psychotherapeutic support services (21). In the United States, a decrease in care for suicide attempts was observed in the early months of the COVID-19 pandemic, followed by an increase in subsequent months among individuals aged 12–25 years (22). This data suggests that the reduction in cases during the coronavirus pandemic years is related to underreporting.

System evaluation showed good data quality; however, it is important to note that completeness was rated poor or very poor for key variables such as gender identity, sexual orientation, recurrence, and place of occurrence. Consistency was rated moderate to low for variables such as type of violence when paired with supplementary information indicating self-inflicted violence, and cases in which the perpetrator’s sex differed from the victim’s. Nonetheless, a gradual improvement in completeness and consistency was observed when comparing 2019 to 2023. 

Data quality may be directly related to a lack of awareness among professionals about the importance and relevance of notifications in the context of public health, as well as to professionals’ demotivation and work overload, which also affects the timely notification and data entry of cases (23).

Completeness was assessed considering mandatory fields and essential fields. Even for mandatory fields such as place of occurrence, gender identity, and sexual orientation, completeness was poor or very poor. The reason is that, despite being mandatory, the field allows invalid information to be included (unknown). This pattern of incompleteness has also been observed in the evaluation of notification completeness for violence against older adults in Niterói, Rio de Janeiro (24), and for violence against children in Pernambuco (25). These findings demonstrate how the violence surveillance system is often neglected in terms of proper notification form completion.

In terms of consistency, it is noteworthy that the classification was low for the variable “type of violence” when paired with supplementary information or any other term indicating self-inflicted violence. According to the Ministry of Health’s guidance document on violence, in cases of self-inflicted violence, the “type of violence” field must be marked as “other,” with the supplementary information indicating self-inflicted violence, since this option is not available among the predefined options in the form (1). However, this field is often completed incorrectly by health professionals, who tend to check more than one type of violence. 

It is necessary to clarify that the guidance stipulates that only the main form of violence at the time of care should be reported (1), but professionals frequently select multiple options. This fact explains why the total number of types of violence reported may exceed the sample size in some studies, such as the one conducted in Niterói on violence against older adults, which covered the period 2011–2020. That study recorded 486 notifications of violence against older adults, but 642 instances of violence were reported (24), which reinforces the understanding that professionals lack knowledge about the correct completion of the notification form.

Representativeness was considered moderate, but some variables related to the individual, such as educational level and marital status, showed low completion rates. It becomes more evident when analyzing victim profiles, as many sensitive fields—for example, sexual orientation, gender identity, and disability/disorder—had high percentages of blank or unknown responses. The absence of such information may negatively impact the provision of care to these individuals, as it is known that the LGBTQIAPN+ population is at higher risk for suicidal behavior when compared to heterosexual and cisgender groups (26–27). 

Although significant improvement was observed, the system was deemed untimely for 24-hour notifications, Sinan data entry within seven days, and same-day case closure. It is important to emphasize that timeliness—particularly regarding case identification and notification within 24 hours of the event—is critical to enable timely follow-up actions. Suicide is often preceded by self-injury and previous attempts, requiring immediate and qualified care to mitigate the risk of death (18). 

Acceptability was deemed inadequate both for 24-hour notification and for proper completion of the essential fields in the notification form. Acceptability has also been evaluated in cases of leptospirosis in Campinas, São Paulo, where the percentage of notifications completed within 24 hours was found to be inadequate (19), as is also the case with self-inflicted violence. Although these are two entirely different conditions, both leptospirosis and self-inflicted violence require immediate action. Low acceptability significantly hinders this process.

While several weaknesses were identified, improvements in timeliness, completeness, and consistency were also observed. These improvements were likely related to the implementation of the action plan established by Resolution No. 7,732/2021 of the Minas Gerais State Health Department (29), amended by Resolution No. 8,384/2022 (30). These resolutions allocated financial resources to municipalities to strengthen surveillance of violence and traffic accidents. Among the various actions to be implemented was the training of health professionals in the municipalities to properly complete notification forms, thereby increasing their sensitivity to identifying cases and completing forms appropriately (29–30).

In conclusion, this study is highly relevant in the context of public health, as it helps identify weaknesses in notification form completion and delays in case identification and reporting, making the system unacceptable for the surveillance of this condition. Efforts should be intensified to improve the surveillance of self-inflicted violence, directing actions toward its preventability and promoting qualified, comprehensive, and equitable care, grounded in gender and diversity policies capable of mitigating the risks of self-harming behavior, especially among the most vulnerable groups.
