# Supplementary material for: Evaluation of the self-inflicted violence surveillance system and characterization of reported cases in the Eastern health macro-region of Minas Gerais, 2019-2023
Source: Epidemiol Serv Saude. 2025 Oct 27;34:e20240754. doi: 10.1590/S2237-96222025v34e20240754.en (PMC12560223; doi:10.1590/S2237-96222025v34e20240754.en)
Supplement: Supplementary Table 1 [file 2237-9622-ress-34-e20240754-supp01-en.pdf]

Supplementary Table 1. Proportion of duplicate records in the Notifiable Health Conditions Information System for reports of self-inflicted violence. Eastern health macro-region, Minas Gerais, 2019–2023.

**Year**	**Notifications (n)**	**Duplicates n (%)**	**Notifications after removing duplicates (n)**
2019	646	6 (0.9)	640
2020	385	4 (1.0)	381
2021	398	5 (1.3)	393
2022	636	23 (3.6)	613
2023	808	18 (2.2)	790
Total	2873	56 (1.9)	2817
