# Supplementary material for: Evaluation of the self-inflicted violence surveillance system and characterization of reported cases in the Eastern health macro-region of Minas Gerais, 2019-2023
Source: Epidemiol Serv Saude. 2025 Oct 27;34:e20240754. doi: 10.1590/S2237-96222025v34e20240754.en (PMC12560223; doi:10.1590/S2237-96222025v34e20240754.en)
Supplement: Tabela suplementar 1 [file 2237-9622-ress-34-e20240754-supp01-pt.pdf]

Tabela suplementar 1. Proporção de registros duplicados no Sistema de Informação de Agravos de Notificação de notificações de violência autoprovocada. Macrorregião de saúde Leste, Minas Gerais, 2019-2023

**Ano**	**Notificações (n)**	**Duplicidades n (%)**	**Notificações após remoção de duplicidades (n)**
2019	646	6 (0,9)	640
2020	385	4 (1,0)	381
2021	398	5 (1,3)	393
2022	636	23 (3,6)	613
2023	808	18 (2,2)	790
Total	2.873	56 (1,9)	2.817
