# Supplementary material for: Evaluation of the self-inflicted violence surveillance system and characterization of reported cases in the Eastern health macro-region of Minas Gerais, 2019-2023
Source: Epidemiol Serv Saude. 2025 Oct 27;34:e20240754. doi: 10.1590/S2237-96222025v34e20240754.en (PMC12560223; doi:10.1590/S2237-96222025v34e20240754.en)
Supplement: Translation

# Translation: Avaliação do sistema de vigilância da violência autoprovocada e caracterização dos casos notificados na macrorregião de saúde Leste de Minas Gerais, 2019-2023

**DOI:** 10.1590/S2237-96222025v34e20240754.pt

**Authors:** Cristian Kelly Costa Santos, Paulo Filipe Silva Siqueira, Waneska Alexandra Alves, Sibele Nascimento Aquino

**Affiliations:** 1 Universidade Federal de Juiz de Fora, Governador Valadares, MG, Brasil

**Keywords:** Sistema de Informação em Saúde, Vigilância em Saúde, Tentativa de Suicídio, Violência, Epidemiologia Descritiva

## Abstract

**Objetivos:**

Analisar as características epidemiológicas das notificações de violência autoprovocada em indivíduos com idade igual ou superior a 10 anos, residentes em municípios da macrorregião de saúde Leste de Minas Gerais no período 2019-2023, e avaliar o sistema de vigilância do agravo por meio dos atributos qualidade dos dados, representatividade, oportunidade e aceitabilidade.

**Métodos:**

Tratou-se de estudo transversal de abordagem descritiva, que analisou o perfil epidemiológico das notificações de violência autoprovocada e os atributos qualidade dos dados, representatividade, oportunidade e aceitabilidade, conforme diretrizes dos Centros de Controle e Prevenção de Doenças dos Estados Unidos.

**Resultados:**

Foram notificados 2.817 casos de violência autoprovocada nessa macrorregião, sendo 69,7% (1.964) em mulheres, com idade mediana de 25 anos. A qualidade dos dados foi classificada como boa (duplicidade aceitável: 1,9%; consistência regular: 83,5%; e completitude regular: 70,9%). A representatividade foi moderada, considerando a completitude do perfil das vítimas (70,1%), o tempo (100,0%) e o local da ocorrência (63,7%). A oportunidade foi inadequada para notificação em 24h (52,1%), digitação em até sete dias (24,8%) e encerramento no mesmo dia (34,2%). A aceitabilidade do sistema foi avaliada como não aceitável, devido à baixa oportunidade e à completitude insatisfatória das variáveis essenciais (68,4%).

**Conclusão:**

Foram identificadas fragilidades no preenchimento das notificações e falta de oportunidade na identificação e notificação dos casos, tornando o sistema não aceitável. Espera-se intensificar estratégias para melhorar a vigilância da violência autoprovocada, direcionando esforços para sua prevenção.

**Aspectos éticos**

A presente pesquisa respeitou os princípios éticos, obtendo os seguintes dados de aprovação:

Comitê de ética em pesquisa: Universidade Federal de Juiz de Fora

Número do parecer: 6.585.259

Data de aprovação: 15/12/2023

Certificado de apresentação de apreciação ética: 74689523.3.0000.5147

Registro do consentimento livre e esclarecido: Dispensado, pois se trata de dados secundários, sendo obtida a dispensa do termo pelo comitê de ética.

## Introdução 

A violência autoprovocada é observada, diariamente, em autolesões ou automutilações leves ou graves, tentativa de suicídio e suicídio consumado (1). Destaca-se como importante problema de saúde pública devido à magnitude dos prejuízos causados às vítimas, aos seus familiares e à sociedade como um todo (2). O agravo é de notificação compulsória no Brasil desde 2011 (3) e notificação imediata desde 2014 (4). 

Houve aumento de notificações e óbitos por violência autoprovocada no Brasil e no mundo. No país, para as notificações, que incluem as tentativas de suicídios, ocorreu o aumento de 743,0% de 2011 para 2019 (5). Esse aumento foi observado entre adolescentes em âmbito escolar, com o acréscimo de 2.800,0% no período 2011-2018 (6). De 2014 a 2018, houve aumento de 19,0% em relação aos óbitos (7). A taxa de mortalidade por suicídio também cresceu entre os adolescentes, tanto no sexo masculino quanto no sexo feminino, com incremento de 81,0% entre 2010 e 2019 (8).

Destaca-se a importância de sistema de vigilância robusto e resiliente que permita a análise consistente da morbimortalidade do agravo, atuando com rigoroso e sistemático processo de coleta de dados, análise e compartilhamento de informações para o alcance dos objetivos para os quais foi implantado (9). Os objetivos do sistema como a identificação e notificação oportuna dos casos, o acompanhamento das vítimas na rede de atenção e a análise do perfil das vítimas e dos fatores de risco devem ser alçados visando subsidiar os gestores na formulação e no aprimoramento de políticas públicas para o enfrentamento desse desafio (10).

Considerando a importância dos sistemas de vigilância em saúde, a avaliação periódica desses sistemas torna-se ação indispensável, pois permite monitorar doenças e agravos relevantes à saúde pública e promove a melhoria na utilização dos recursos públicos, tornando o sistema mais eficaz e eficiente. Além disso, permite verificar se a vigilância da doença, do agravo ou do evento de saúde atende aos objetivos para os quais ele foi implantado. A avaliação do sistema ocorre por meio de atributos que refletem a robustez e a confiabilidade dos dados, organizados em dois blocos: quantitativos (sensibilidade, valor preditivo positivo, representatividade e oportunidade) e qualitativos (simplicidade, flexibilidade, estabilidade, qualidade dos dados e aceitabilidade) (9). 

Este estudo se justifica pela relevância da violência autoprovocada no cenário nacional e local, com aumento de casos em todas as faixas etárias e estratos sociais. Desse modo, conhecer como funcionam os atributos do sistema de vigilância da violência autoprovocada no Leste de Minas Gerais é fundamental para garantir eficácia e precisão nas ações de prevenção e intervenção. 

Os objetivos deste estudo foram analisar as características epidemiológicas das notificações de violência autoprovocada de indivíduos com idade de 10 anos ou mais, residentes em municípios localizados na macrorregião de saúde Leste de Minas Gerais no período 2019-2023, e avaliar o sistema de vigilância do agravo por meio dos atributos qualidade dos dados, representatividade, oportunidade e aceitabilidade.

## Métodos 

### Desenho do estudo

Tratou-se de estudo transversal, de abordagem descritiva, que analisou o perfil epidemiológico das notificações de violência autoprovocada e os atributos de qualidade dos dados, representatividade, oportunidade e aceitabilidade, conforme diretrizes dos Centros de Controle e Prevenção de Doenças (Centers for Disease Control and Prevention – CDC) dos Estados Unidos (9). Foram analisadas notificações de indivíduos residentes na macrorregião Leste de Minas Gerais, registradas no Sistema de Informação de Agravos de Notificação (Sinan) entre 2019 e 2023. Cumpre esclarecer que o período de estudo foi definido considerando a análise de dados antes, durante e pós-pandemia da covid-19.

### Local do estudo

Minas Gerais é uma unidade federada localizada no Sudeste do Brasil e possui 16 macrorregiões de saúde. É o primeiro estado do país em número de municípios e o segundo em número de habitantes (11). A macrorregião de saúde Leste de Minas Gerais possui 51 municípios, distribuídos em quatro microrregiões, cujos municípios polo são Governador Valadares, Mantena, Resplendor, Peçanha/São João Evangelista/Santa Maria do Suaçuí (12), com população informada em 2022 de 643.031 habitantes. A média populacional por município, excluindo Governador Valadares, é 7.717 habitantes, tratando-se majoritariamente de municípios de pequeno porte. Governador Valadares é a sede da macrorregião, comporta os serviços de saúde de maior complexidade e possui população de 257.172 habitantes (11).

### Fonte de dados

Foram utilizados dados nominais secundários do Sinan, de indivíduos com 10 anos ou mais, disponibilizados pela Superintendência Regional de Saúde de Governador Valadares em março de 2024, o que garantiu dados atualizados em tempo real. Essa unidade regional pertence à Secretaria de Estado de Saúde de Minas Gerais e é responsável pela estrutura organizacional de saúde na macrorregião. 

O Sinan funciona de forma descentralizada para os municípios. Todos os serviços de saúde são unidades notificadoras e os registros das notificações entraram na base nacional de vigilância do agravo. Foram incluídos apenas os registros notificados com suspeita ou confirmação de lesão autoprovocada (tentativa de suicídio ou automutilação), conforme as normativas vigentes do Ministério da Saúde (1). A população do estudo foi definida pelo apontamento da dificuldade em se determinar a intencionalidade da autolesão em crianças menores de 10 anos (13). 

### Variáveis do estudo

Na etapa descritiva, foram analisadas as variáveis sociodemográficas: sexo (masculino, feminino), faixa etária em anos (10-19, 20-34, 35-49, 50-64, 65-79, ≥80), raça/cor da pele (branca, preta/parda, amarela, indígena, ignorado/vazio), escolaridade em anos de estudo (<4; 4-7; 8-10; ≥11, ignorado/vazio), situação conjugal (solteiro, casado/união consensual, viúvo, separado, não se aplica, ignorado/vazio), orientação sexual (heterossexual, homossexual, bissexual, não se aplica, ignorado/vazio), identidade de gênero (travesti, mulher transsexual, homem transsexual, não se aplica, ignorado/vazio) e presença de algum tipo de deficiência/transtorno (sim, não, ignorado/vazio). 

Para a análise da ocorrência, analisaram-se as variáveis: local de ocorrência (residência, via pública, outros, ignorado/vazio), ocorreu outras vezes (sim, não, ignorado/vazio), meio de agressão (enforcamento, envenenamento/intoxicação, objeto perfuro/cortante, força corporal/espancamento, objeto contundente, substância ou objeto quente, ameaça, arma de fogo, outros) e encaminhamento para rede de saúde (sim, não, ignorado/vazio). As variáveis deste estudo foram definidas pela sua relevância epidemiológica, conforme recomendações do Ministério da Saúde (14).

Para a abordagem avaliativa, foram estudados os atributos a seguir (Tabela 1).

**Tabela 1:** Parâmetros utilizados para avaliação dos atributos de qualidade dos dados, representatividade, oportunidade e aceitabilidade

Atributo	Itens para avaliação	Classificação	Fórmula de cálculo	Classificação geral
**Qualidade dos dados**	Duplicidade	Duplicidade aceitável (A) <5,0%, não aceitável (NA) ≥5,0%	Média percentual=n duplicidades*100/n notificações	Ótima (duplicidade A, completitude E, consistência E); **Boa** (duplicidade NA, completitude E/B/ Re/, inconsistência E/Re; duplicidade A/NA, completitude B/ Re/R, inconsistência E/Re/B; duplicidade A/NA, completitude E/B/ Re/R/MR, inconsistência Re/R); **Ruim** (duplicidade NA, completitude R/MR, inconsistência B).
	Completitude	Excelente (E)≥95,0%, boa (B) entre 90,0 e 95,0%, regular (Re) 70,0 a 89,0%, ruim (R) 50,0 a 69,0% e muito ruim (MR)<50,0%	Média percentual=soma do percentual de completitude das variáveis/n de variáveis	
	Consistência	Excelente (E)≥90,0%, regular (Re)≥70,0 e <90,0% ou baixa (R) <70,0%	Média percentual=soma do percentual de consistência das variáveis/n de variáveis	
Representatividade	Pessoa (perfil das vítimas)	Representativo (R): ≥70,0%; pouco representativo (PR) <70,0%	Soma do percentual de completitude das variáveis/n de variáveis	Representativo (pessoa, tempo e lugar=R); **Representatividade moderada** (dois resultados R+um PR); **Pouco representativo** (pessoa, tempo e lugar=PR ou um R e dois PR).
	Tempo (data da ocorrência)		Percentual de completitude da variável data da ocorrência	
	Lugar (local da ocorrência)		Percentual de completitude do local de ocorrência	
Oportunidade	Notificação em até 24h a partir da data da ocorrência.	Oportuno (OP): ≥90,0% Inoportuno (IN): <90,0%	N notificações em até 24h*100/n notificações	Oportuno (notificação em até 24h, digitação em sete dias e encerramento no mesmo dia da notificação=OP); Oportuno (dois resultados O e um resultado IN); **Inoportuno** (notificação em até 24h, digitação em sete dias e encerramento no mesmo dia da notificação IN ou um OP e dois IN.
	Digitação no Sinan até sete dias após a notificação		N notificações digitadas em até sete dias*100/n notificações	
	Encerramento na mesma data da notificação		N notificação encerradas no mesmo dia da notificação*100/n notificações	
Aceitabilidade	Notificação em tempo oportuno	Aceitável (A): ≥70,0% Não aceitável: <70,0%	N notificações em até 24h*100/n notificações	Aceitável (notificação em tempo oportuno e percentual de completitude dentro do parâmetro de aceitabilidade=A); Aceitável (um A e um NA=A); **Não aceitável** (notificação em tempo oportuno e percentual de completitude=NA).
	Completitude de oito variáveis		Média percentual=soma do percentual de completitude das variáveis/n de variáveis

- Qualidade dos dados estava relacionada a dados válidos com informações completas e precisas, considerando três dimensões: completitude, consistência e não duplicidades (9).
- Completitude fez referência à proporção dos campos obrigatórios e essenciais da ficha de notificação preenchidos com informações válidas, ou seja, ausência de dados em branco ou marcados como ignorado. Foram avaliadas as variáveis sociodemográficas e de ocorrência dos casos, incluindo ocupação e gestante. Os parâmetros adotados para avaliar a completitude variaram de “excelente” a “muito ruim” (15).
- Consistência foi a conformidade entre os diferentes campos de registro ou conjunto de dados em um sentido lógico. Os parâmetros adotados para avaliar consistência variaram de “excelente” a “baixa” (16). Avaliaram-se as combinações de variáveis: sexo masculino e campo gestante preenchido com “não se aplica”; data de nascimento compatível com a idade da vítima; lesão autoprovocada e meio de agressão diferente de ameaça; sexo do autor divergente do sexo da vítima; grau de parentesco do agressor com a vítima “a própria pessoa”; lesão autoprovocada e tipo de violência com opção preenchida “outros” com a informação complementar de violência autoprovocada, ou qualquer outro termo ou expressão que indicasse esse tipo de violência; data da notificação igual ou posterior à ocorrência.
- Duplicidade foi quando o mesmo caso foi registrado mais de uma vez no sistema de vigilância. A análise de duplicidade envolveu a detecção e remoção de registros duplicados. Foram considerados como sendo a mesma pessoa aqueles indivíduos com o mesmo nome completo, data de nascimento, sexo, idade, nome da mãe, data da ocorrência e local da ocorrência e do campo observação (1). O critério utilizado para considerar a duplicidade aceitável foi o mesmo definido por Abath et al. (17).
- Representatividade foi a capacidade de os dados coletados permitirem a descrição do agravo em pessoa, tempo e lugar (9). Para avaliar esse atributo, foram estudadas as variáveis relacionadas ao perfil das vítimas (sexo, idade, escolaridade, raça/cor da pele, situação conjugal, identidade de gênero e orientação sexual), ao ano e ao local da ocorrência.
- Oportunidade fez referência ao tempo em que as informações do evento estavam disponíveis para quem precisasse, a fim de desencadear ações necessárias ao seu controle (9). A notificação de violência autoprovocada à vigilância epidemiológica municipal deveria ser imediata (em até 24h após a ocorrência), assim como a digitação no Sinan deveria ocorrer em até sete dias. Os casos foram encerrados no momento que a notificação foi preenchida (1,4,7).
- Aceitabilidade foi como os profissionais e as instituições participaram do sistema de vigilância e desejaram colaborar e utilizar o sistema para implementação de ações imediatas para mitigar os riscos de recorrência (9,18). Foram avaliadas a notificação em tempo oportuno (até 24h) e as informações relevantes para a condução do caso, como: raça/cor da pele, escolaridade, transtorno/deficiência, orientação sexual, identidade de gênero, se ocorreu outras vezes, local da ocorrência, meio de agressão e se foi encaminhado à rede de saúde. Considerou-se aceitável quando as médias dos parâmetros de tempo e completitude foram maiores ou igual a 70,0% (19).

### Viés 

Para minimizar possíveis vieses de seleção e de informação, os registros utilizados foram aqueles notificados no Sinan como tentativa de suicídio ou automutilação intencional, seguindo critérios padronizados pelo Ministério da Saúde. Registros com informações incompletas ou inconclusivas sobre a intencionalidade do evento foram excluídos para preservar a qualidade e confiabilidade dos dados analisados. Para assegurar a representatividade populacional e minimizar o viés geográfico, apenas notificações de municípios pertencentes à macrorregião de saúde Leste de Minas Gerais foram incluídas. 

### Métodos estatísticos

Foram conduzidas análises descritivas exploratórias com cálculos de frequências absoluta (n) e relativa (%) e análises inferenciais com aplicação dos testes U de Mann-Whitney, para idade, e qui-quadrado de Pearson, para verificar associações entre as variáveis categóricas de interesse na população estudada. Os softwares Microsoft Excel e Jamovi (v.2.4.8) foram utilizados nas análises, ao nível de significância de 5,0% (p-valor<0,05). Após as análises, os dados foram anonimizados e disponíveis em repositório em: https://doi.org/10.48331/scielodata.OGYNWG (20).

## Resultados 

De 2019 a 2023, foram notificados 2.873 casos de violência autoprovocada em pessoas com 10 anos ou mais, residentes em municípios da macrorregião Leste de Minas Gerais. Após exclusão de 56 (1,9%) registros duplicados, foram avaliadas 2.817 notificações (Tabela suplementar 1). A série histórica revelou queda nas notificações nos anos pandêmicos de 2020 (n=381) e 2021 (n=393), com redução de 40,5% e 38,6% em comparação a 2019 (n=640).

A idade mediana das vítimas foi 25 anos, amplitude de 10-91 anos, com predomínio nos adultos jovens, na faixa etária 10-34 anos (69,9%; n=1.968; p-valor<0,001). A maioria das vítimas era do sexo feminino (69,7%; n=1.964; p-valor<0,001) e se autodeclarava preta/parda (74,9%; n=2.111; p-valor=0,071), Em relação à ocorrência, as diferenças observadas foram para local de ocorrência, com predomínio no domicílio (59,7%; n=1.681; p-valor<0,001), e meio de agressão – por enforcamento para homens (13,8%; n=118; p-valor<0,001) e envenenamento para mulheres (62,5%; n=1.228; p-valor<0,001) (Tabela 2).

**Tabela 2:** Características dos casos de violência autoprovocada notificados no Sistema de Informação de Agravos de Notificação separados por sexo. Macrorregião de saúde Leste, Minas Gerais, 2019-2023

Variáveis	Total n (%)	Masculino n (%)	Feminino n (%)	p-valor
	2.817 (100,0)	853 (30,3)	1.964 (69,7)	
Variáveis sociodemográficas				
**Faixa etária** (anos)				<0,001
10-19	879 (31,2)	207 (24,3)	672 (34,2)	
20-34	1.089 (38,7)	362 (42,4)	727 (37,3)	
35-49	594 (21,1)	168 (19,7)	426 (21,7)	
50-64	194 (6,9)	78 (9,1)	116 (5,9)	
65-79	52 (1,8)	30 (3,5)	22 (1,1)	
≥80	9 (0,3)	8 (0,09)	1 (0,1)	
**Raça/cor da pele**				0,071
Branca	511 (18,1)	130 (15,2)	381 (19,4)	
Preta/parda	2.111 (74,9)	656 (76,9)	1.455 (74,1)	
Amarelo	37 (1,3)	12 (1,4)	25 (1,3)	
Indígena	8 (0,8)	2 (0,2)	6 (0,3)	
Ignorado/vazio	150 (5,3)	54 (6,2)	97 (4,9)	
**Escolaridade** (anos)				<0,001
<4	80 (2,8)	33 (3,9)	47 (2,4)	
4-7	271 (9,6)	75 (8,8)	196 (10,0)	
8-10	406 (14,4)	110 (12,9)	296 (15,1)	
≥11	372 (13,2)	86 (10,6)	286 (14,6)	
Ignorado/vazio	1.688 (59,9)	549 (64,4)	1.139 (58,0)	
**Situação conjugal**				0,559
Solteiro	1.152 (40,9)	359 (42,1)	793 (40,4)	
Casado/união consensual	504 (17,9)	137 (16,1)	367 (18,7)	
Viúvo	20 (0,7)	4 (0,5)	16 (0,8)	
Separado	87 (30,1)	26 (3,0)	61 (3,1)	
Não se aplica	31 (1,1)	10 (1,2)	21 (1,1)	
Ignorado/vazio	1.023 (36,3)	317 (37,1)	706 (35,9)	
**Orientação sexual**				0,167
Heterossexual	1.196 (42,5)	338 (39,6)	858 (43,7)	
Homossexual	50 (1,8)	20 (2,3)	30 (1,5)	
Bissexual	19 (0,7)	5 (0,6)	14 (0,7)	
Não se aplica	99 (3,5)	28 (3,3)	71 (3,6)	
Ignorado/vazio	1.453 (51,6)	462 (54,2)	991 (50,5)	
**Identidade de gênero**				0,002
Travesti	6 (0,2)	6 (0,7)	0 (0,0)	
Mulher transsexual	14 (0,5)	4 (0,5)	10 (0,5)	
Homem transsexual	0 (0,0)	0 (0,0)	0 (0,0)	
Não se aplica	1.208 (42,9)	351 (41,1)	877 (43,6)	
Ignorado/vazio	1.589 (46,4)	492 (57,7)	1.097 (55,9)	
**Deficiência ou transtorno**				0,156
Sim	880 (31,2)	268 (31,4)	612 (31,2)	
Não	942 (33,4)	262 (30,7)	680 (34,6)	
Ignorado/vazio	995 (35,3)	323 (37,9)	672 (34,2)	
Variáveis de ocorrência				
**Local de ocorrência**				<0,001
Residência	1.681 (59,7)	447 (52,4)	1.234 (62,8)	
Via pública	120 (4,3)	50 (5,9)	70 (3,6)	
Outros	136 (4,8)	67 (7,9)	69 (3,5)	
Ignorado/vazio	880 (31,2)	289 (33,9)	591 (30,1)	
**Ocorreu outras vezes**				<0,001
Sim	945 (33,5)	220 (25,8)	725 (36,9)	
Não	756 (26,8)	266 (31,2)	490 (24,9)	
Ignorado/vazio	1.116 (39,6)	367 (43,0)	749 (38,1)	
**Meio de agressão**				
Enforcamento	194 (6,9)	118 (13,8)	76 (3,9)	<0,001
Envenenamento/intoxicação	1.655 (58,8)	427 (50,1)	1.228 (62,5)	<0,001
Objeto perfuro/cortante	516 (18,3)	140 (16,4)	376 (19,1)	0,188
Força corporal/espancamento	205 (7,3)	73 (8,6)	132 (6,7)	0,181
Objeto contundente	67 (2,4)	20 (2,3)	47 (2,4)	0,918
Substância ou objeto quente	35 (1,2)	9 (1,1)	26 (1,3)	0,724
Ameaça	59 (2,1)	17 (2,0)	42 (2,1)	0,862
Arma de fogo	18 (0,6)	9 (1,1)	9 (0,5)	0,144
Outros	262 (9,3)	81 (9,5)	181 (9,2)	0,572
**Encaminhamento** (**Rede de Saúde**)				0,793
Sim	2.556 (90,7)	770 (90,3)	1.786 (90,9)	
Não	233 (8,3)	75 (8,8)	158 (8,0)	
Ignorado/vazio	28 (1,0)	8 (0,9)	(1,0)	

^a^Teste U de Mann-Whitney; ^b^Teste qui-quadrado de Pearson.

A qualidade dos dados foi avaliada considerando as dimensões de completitude, duplicidade e consistência. A completitude foi avaliada como excelente e boa para as variáveis sexo, idade, encaminhamento para rede de saúde, meio de agressão e raça/cor da pele (Tabela 3). Para duplicidade, 2019 foi o ano com menor percentual de duplicidades (0,9%; n=6), e 2022, com maior percentual (3,6%; n=23) (Tabela suplementar 1). 

**Tabela 3:** Completitude dos campos da ficha de notificação de violência autoprovocada. Macrorregião de saúde Leste, Minas Gerais, 2019-2023

Variáveis	2019	2020	2021	2022	2023	Total (n=2.817) %	Classificação
	(n=640) %	(n=381) %	(n=393) %	(n=613) %	(n=790) %		
Obrigatórias							
Sexo	100,0	100,0	100,0	100,0	100,0	100,0	Excelente
Idade	100,0	100,0	100,0	100,0	100,0	100,0	Excelente
Encaminhamento para rede de saúde	99,7	99,7	100,0	98,5	98,0	99,0	Excelente
Gestante	62,3	84,3	77,9	86,0	87,8	79,8	Regular
Local de ocorrência	58,8	63,3	58,3	71,1	82,9	68,8	Ruim
Orientação sexual	41,1	48,0	38,2	50,1	58,4	48,4	Muito ruim
Identidade de gênero	32,5	43,8	33,4	51,9	51,4	43,6	Muito ruim
Essenciais							
Meio de agressão	90,3	97,4	97,3	95,0	96,5	95,6	Excelente
Raça/cor da pele	93,4	97,6	88,5	94,3	97,6	94,7	Bom
Situação conjugal	64,0	59,8	52,7	61,3	72,5	63,7	Ruim
Deficiência/ transtorno	43,8	50,9	54,7	64,8	93,2	64,8	Ruim
Ocorreu outras vezes	55,5	56,7	52,7	58,4	71,5	60,4	Ruim
Escolaridade	35,3	34,4	28,0	41,6	51,6	40,1	Muito ruim
Ocupação	31,6	27,3	21,6	36,3	43,9	34,1	Muito ruim

A consistência avaliou a coerência entre sete variáveis relacionadas, sendo cinco classificadas como excelente (sexo masculino e campo gestante preenchido com “não se aplica”; data da notificação igual ou posterior à ocorrência; data de nascimento compatível com a idade da vítima; sim para lesão autoprovocada e grau de parentesco do agressor com a vítima, a própria pessoa; e meio de agressão com informação válida divergente de ameaça) (Tabela 4). Na análise global, a média percentual e a sua classificação, nos cinco anos avaliados, foram: duplicidade 1,9%, aceitável; consistência 83,5%, regular; e completitude 70,9%, regular. A qualidade dos dados foi classificada como boa.

**Tabela 4:** Consistência entre as variáveis relacionadas da ficha de notificação de violência autoprovocada. Macrorregião de saúde Leste, Minas Gerais, 2019-2023

Variáveis	2019	2020	2021	2022	2023	Total (n=2.817) %	Classificação
	(n=640) %	(n=381) %	(n=393) %	(n=613) %	(n=790) %		
Sexo masculino e campo gestante preenchido com não se aplica	100,0	100,0	100,0	100,0	100,0	100,0	Excelente
Data da notificação igual ou posterior à ocorrência	100,0	100,0	100,0	100,0	100,0	100,0	Excelente
Data de nascimento compatível com a idade da vítima	96,9	96,3	95,4	97,6	97,6	97,0	Excelente
Grau de parentesco do agressor com a vítima, a própria pessoa	92,2	87,9	90,4	87,4	92,4	90,5	Excelente
Meio de agressão com informação válida divergente de ameaça	88,0	95,0	97,2	92,7	94,8	93,1	Excelente
Sexo do autor divergente do sexo da vítima	90,3	86,3	84,7	85,5	90,0	87,9	Regular
Tipo de violência com informação complementar de violência autoprovocada	6,7	9,7	6,6	11,3	35,2	16,1	Baixa

A avaliação dos dados indicou que o sistema é representativo para pessoa, com percentual médio de completitude das variáveis relacionadas ao perfil das vítimas de 70,1% e tempo (data da ocorrência) de 100,0%. Considerando o local onde o evento ocorreu, foi pouco representativo com percentual de completitude de 63,7%. A representatividade do sistema foi moderada.

Cabe ressaltar que, em relação ao lugar, considerando o município de ocorrência, destacou-se a distribuição na macrorregião por município de 98,0% (n=50), visto que apenas um município não registrou nenhuma ocorrência de violência autoprovocada nesse período. Observou-se aumento na distribuição das ocorrências por municípios, pois 84,3% (n=43) dos municípios registraram casos em 2019; em 2023, subiu para 94,1% (n=48). 

A oportunidade do sistema foi classificada como inadequada para notificação em 24h, digitação em sete dias e encerramento no mesmo dia da notificação (Tabela 5). A mediana da notificação dos casos foi um dia (0-4.778 dias); da digitação no Sinan, 16 dias (0-4.781 dias); e do encerramento dos casos, três dias (0-735 dias). 

**Tabela 5:** Avaliação da oportunidade dos casos de violência autoprovocada notificados no Sistema de Informação de Agravos de Notificação (Sinan). Macrorregião de saúde Leste, Minas Gerais, 2019-2023

Variáveis	2019	2020	2021	2022	2023	Total	
	(n=640) %	(n=381) %	(n=393) %	(n=613) %	(n=790) %	(n=2.817) %	p-valor
Casos notificados até 24h após a ocorrência	35,0	50,9	45,8	59,1	64,4	52,1	<0,001
Casos digitados no Sinan em até sete dias após a ocorrência	16,3	25,5	14,2	32,8	30,6	24,8	<0,001
Casos encerrados no mesmo dia da notificação	28,0	37,0	34,9	32,0	39,6	34,2	<0,001

^a^Teste qui-quadrado de Pearson.

O sistema também foi classificado como não aceitável para identificação e notificação dos casos em tempo oportuno, visto que 52,1% (n=1.469) ocorreram em até 24h. Observou-se a completude percentual média de 68,4% para as variáveis ​​consideradas essenciais à condução e ao manejo das vítimas (raça/cor da pele, escolaridade, presença de transtorno/deficiência, orientação sexual, identidade de gênero, recorrência da ocorrência, local da ocorrência, meio de agressão e encaminhamento à rede de saúde). 

## Discussão 

Os resultados apontaram algumas fortalezas e fragilidades do sistema de vigilância macrorregional da violência autoprovocada. Ele apresentou boa qualidade dos dados e representatividade moderada, porém foi inoportuno para todas as variáveis de tempo avaliadas e não aceitável por parte dos profissionais de saúde, uma vez que a identificação e a notificação em tempo oportuno, uma das premissas essenciais de qualquer sistema de vigilância, não foram atendidas na maioria dos casos.

Cumpre ressaltar que trabalhar com banco de dados secundário pode apresentar certas limitações, a exemplo de subnotificações, ausência de informações relevantes para o estudo e preenchimento inadequado da ficha de notificação. Outros limitadores foram a avaliação de apenas quatro atributos de nove recomendados pelo CDC (9) e a pandemia da covid-19, já que houve redução importante no número de notificações nesse período. Apesar das limitações, os resultados aqui apresentados foram robustos, no sentido de apresentar o panorama geral da vigilância da violência autoprovocada na macrorregião, com análise dos dados ano a ano, permitindo a avaliação antes, durante e após a pandemia. 

A pandemia da covid-19 impactou sobremaneira as notificações, ficando evidente o quadro de subnotificação nos anos pandêmicos. Esse período foi marcado por severas restrições e imposição de isolamento social para controle da doença. Os serviços de saúde ficaram sobrecarregados e foram obrigados a priorizar o atendimento aos pacientes com quadros mais graves, o que pode ter dificultado o acesso a alguns serviços ambulatoriais e de apoio psicoterapêutico (21). Nos Estados Unidos, foi observada redução em atendimentos por tentativa de suicídio nos primeiros meses da pandemia, seguido de aumento nos meses subsequentes entre pessoas de 12-25 anos (22). Isso sugere que a redução dos casos nos anos pandêmicos está relacionada à subnotificação.

A avaliação do sistema apresentou boa qualidade dos dados, mas é preciso destacar que a completitude foi avaliada como ruim ou muito ruim para variáveis ​​importantes, como identidade de gênero, orientação sexual, recorrência e local de ocorrência. A consistência foi regular e baixa nos relacionamentos das variáveis tipo de violência com informação complementar de violência autoprovocada e sexo do autor divergente do sexo da vítima. No entanto, destaca-se melhora gradativa na completitude e na consistência, quando se compara 2019 com 2023. 

A qualidade dos dados pode estar relacionada diretamente à falta de conhecimento, por parte do profissional, sobre a importância e relevância da notificação no contexto da saúde pública, além da desmotivação e sobrecarga de trabalho desses profissionais, o que também reflete na notificação e digitação dos casos em tempo oportuno (23)

A completitude foi avaliada considerando campos obrigatórios e campos essenciais, mesmo para campos obrigatórios como local de ocorrência, identidade de gênero e orientação sexual, a completitude foi ruim ou muito ruim. Isso ocorre porque o campo, apesar de ser obrigatório, permite incluir informação não válida (ignorada). Esse padrão de incompletude foi observado na avaliação da completitude das notificações de violência contra pessoas idosas em Niterói, Rio de Janeiro (24), e para violência perpetrada contra crianças em Pernambuco (25). Isso demonstra o quanto o sistema de vigilância da violência é negligenciado em relação ao preenchimento das notificações.

Para consistência, chama atenção a classificação baixa para a variável tipo de violência com informação complementar de violência autoprovocada ou qualquer outro termo ou expressão que a caracterize. O instrutivo de violência do Ministério da Saúde determina que, em casos de violência autoprovocada, o campo tipo de violência deve ser marcado “outros” com informação complementar de violência autoprovocada, pois a ficha não traz essa alternativa a ser marcada no tipo de violência (1). Contudo, esse campo da ficha de notificação (tipo de violência) vem sendo preenchido de forma equivocada pelos profissionais de saúde quando marcam mais de uma opção. 

Faz-se necessário esclarecer que o instrutivo prevê que apenas a violência principal no momento do atendimento deve ser informada (1), porém os profissionais acabam marcando mais de uma alternativa. Isso explica o total do tipo de violência ser maior que a amostra em alguns estudos, como o conduzido em Niterói, sobre violência contra idosos, cujo período foi 2011-2020. O número de notificações de violência contra idosos foi 486, todavia o número de tipos de violência registrados nas notificações foi 642 (24). Isso reforça o entendimento da falta de conhecimento dos profissionais sobre o preenchimento correto da ficha de notificação.

A representatividade foi moderada, mas chama atenção algumas variáveis relacionadas à pessoa como escolaridade e situação conjugal com baixo preenchimento. Isso fica mais evidente quando se analisa o perfil das vítimas, dado que muitos campos sensíveis como orientação sexual, identidade de gênero e deficiência/transtorno apresentaram elevado percentual de informações ignoradas ou em branco. A ausência de tais informações pode interferir negativamente na assistência a essas pessoas, pois é sabido que a população LGBTQIAPN+ têm maior risco para o comportamento suicida quando comparado a grupos heterossexuais e cisgêneros (26-27). 

Apesar de apresentar melhora significativa, o sistema foi avaliado como inoportuno para notificação em até 24h, digitação no Sinan em até sete dias e encerramento no mesmo dia da notificação. Cabe ressaltar que a oportunidade, principalmente relacionada à identificação e à notificação em até 24h a partir da ocorrência, é extremamente relevante para a adoção de medidas de acompanhamento dessas vítimas. O suicídio é muitas vezes precedido de autoagressões e tentativas prévias, necessitando de escuta qualificada e urgente do indivíduo para mitigar o risco do óbito (18). 

A aceitabilidade foi inadequada tanto para a notificação em até 24h quanto para o preenchimento correto dos campos essenciais da ficha de notificação. A aceitabilidade também foi avaliada em casos de leptospirose em Campinas, São Paulo, onde foi verificado um percentual de notificações em até 24h inadequado (19), assim como ocorre na violência autoprovocada. Mesmo sendo dois agravos totalmente distintos, a leptospirose e a violência autoprovocada precisam que ações sejam tomadas de forma imediata, contudo essa baixa aceitabilidade interfere demasiadamente nesse contexto.

Algumas fragilidades foram apresentadas, mas destaca-se melhora na oportunidade, completitude e consistência. Possivelmente, essa melhora esteve relacionada à implementação do plano de ação previsto na Resolução nº 7.732/2021, da Secretaria de Estado de Saúde de Minas Gerais (29), alterada pela Resolução nº 8.384/2022 (30). Essas resoluções disponibilizaram recursos financeiros para os municípios fortalecerem a vigilância das violências e dos acidentes de trânsito. Entre as várias ações que devem ser implementadas, uma delas foi capacitar os profissionais da rede de saúde dos municípios quanto ao preenchimento da ficha de notificação, tornando-os mais sensíveis à identificação dos casos e ao preenchimento adequado da ficha de notificação (29-30).

Em conclusão, destaca-se o quão relevante é este estudo no contexto da saúde pública, na medida em que permite identificar fragilidades no preenchimento das notificações, falta de oportunidade na identificação e notificação dos casos, tornando o sistema não aceitável para vigilância do agravo. Espera-se intensificar estratégias para melhorar a vigilância da violência autoprovocada, direcionando esforços para sua evitabilidade e promovendo assistência qualificada, integral e equitativa, pautada em políticas de gênero e diversidade que possam mitigar os riscos para o comportamento autolesivo, especialmente nos grupos mais vulneráveis.

## Material Suplementar

Tabela suplementar 1
